# Core Set of Responsive and Discriminatory Measures for Use in Pragmatic Trials of Youth With Axial Juvenile Spondyloarthritis

**DOI:** 10.1002/acr.25565

**Published:** 2025-06-23

**Authors:** Timothy G. Brandon, Rui Xiao, Daniel J. Lovell, Edward Oberle, Matthew L. Stoll, Nancy A. Chauvin, Michael L. Francavilla, Walter P. Maksymowych, Pamela F. Weiss

**Affiliations:** ^1^ Children's Hospital of Philadelphia Philadelphia Pennsylvania; ^2^ Perelman School of Medicine University of Pennsylvania, and Children's Hospital of Philadelphia; ^3^ Cincinnati Children's Hospital Medical Center and University of Cincinnati Cincinnati Ohio; ^4^ Nationwide Children's Hospital and The Ohio State University Columbus; ^5^ University of Alabama at Birmingham; ^6^ The Imaging Institute, The Cleveland Clinic Cleveland Ohio; ^7^ Whiddon College of Medicine University of South Alabama Mobile; ^8^ University of Alberta and CARE Arthritis Edmonton Alberta Canada

## Abstract

**Objective:**

The objective of this study was to determine a core set of measures for youth with juvenile spondyloarthritis and axial disease (axJSpA), using the juvenile arthritis working group Outcome Measures in Rheumatology framework.

**Methods:**

This was a prospective multicenter study of youth with axJSpA. Participants (aged 8–18 years) all initiated tumor necrosis factor inhibitor (TNFi) therapy and completed questionnaires, examinations, and magnetic resonance imaging (MRI) at baseline and 12 weeks. Responsiveness and discrimination were assessed using standardized response mean (SRM) and standardized mean difference (SMD). For highly correlated (r > |0.80|) items within domains, larger SRM and SMD were prioritized, and minimal clinically important improvement was determined for each.

**Results:**

Of the evaluable cohort (N = 57), 68.4% were male, and the median age was 15.3 years; 70.2% of youth treated with TNFi had clinical response (change ≥2 in patient global assessment). Although 58% had continued MRI inflammation, 77% of those patients reported moderate clinical improvement. The final axJSpA core set contained the following: Patient‐Reported Outcomes Measurement Information System (PROMIS) pain interference (SRM 0.77, SMD 0.5), the sacroiliac joint inflammation score (SRM 1.02, SMD 0.52), PROMIS mobility (SRM 0.83, SMD 0.75), and patient global well‐being (SRM 0.88, SMD not applicable). All overall and composite disease activity measures tested, except the physician global assessment, had high SRM and SMD. Subgroup analysis demonstrated differences by biologic sex and overweight status. Improvement in the MRI inflammation score was greater in male patients. Improvement in the PROMIS pain interference and mobility measures was greater in those with normal body mass index.

**Conclusion:**

A set of measures was developed for youth with axJSpA.

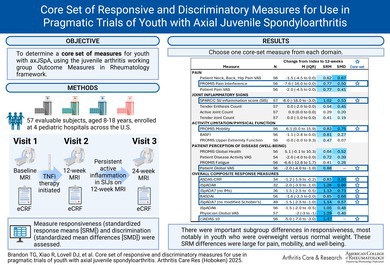

## INTRODUCTION

Approximately 15% to 25% of youth with arthritis have juvenile spondyloarthritis (JSpA) and approximately 20% of youth with JSpA have axial disease (axJSpA).^1^ Even though sacroiliitis is a feature of JSpA that does not respond to standard first‐line juvenile idiopathic arthritis (JIA) therapy, namely disease modifying antirheumatic drugs such as methotrexate, there is only one published clinical trial focused on youth with axial arthritis, which was ultimately a negative study perhaps due in part to the use of SpA outcomes validated in adults but not youth.^2^ Despite the negative trial results, most pediatric rheumatologists firmly believe tumor necrosis factor inhibitor (TNFi) therapies are effective for axial arthritis, as evidenced by (1) an unpublished case‐based survey of voting members of the Childhood Arthritis Rheumatology and Research Association (N = 369) in April 2015, which demonstrated that the use of TNFi therapy was >10 times higher for clinical scenarios of youth with peripheral arthritis and sacroiliitis compared to those with peripheral arthritis only (66% vs 6%; *P* < 0.001), and (2) inclusion of TNFi therapy as first‐line therapy (after nonsteroidal anti‐inflammatory drugs) in the American College of Rheumatology (ACR)/Arthritis Foundation treatment guidelines for sacroiliitis in youth with juvenile arthritis.^2^

**SIGNIFICANCE & INNOVATIONS**

- With many available assessment tools, including patient‐reported outcomes, physician‐based assessment, and imaging tools, there are no data comparing performance of outcome measures in youth with axial disease and juvenile spondyloarthritis (axJSpA) to facilitate identification of which to measure in pragmatic trials or routine clinical care.
- The most responsive and discriminative measures for youth with axJSpA in their Outcome Measures in Rheumatology domains were Patient‐Reported Outcomes Measurement Information System (PROMIS) pain interference, the magnetic resonance imaging sacroiliac joint inflammation score, PROMIS mobility, and the patient global well‐being visual analog scale.
- There were important subgroup differences in responsiveness, most notably in youth who were overweight versus normal weight. These standardized response mean differences were large for pain, mobility, and well‐being.
- Selection of measures should take anticipated baseline disease activity, body mass index, and disease manifestations (enthesitis, peripheral arthritis) of the target population into account.

Current JIA trials are conducted primarily in youth with polyarticular disease (five or more joints) or with a peripheral joint course affecting a minimum of three joints. In a recent international cohort of youth with axJSpA, only 54% had peripheral arthritis and 11.4% had polyarticular course arthritis.^3^ Given the recent advances in novel therapies for adults with SpA, it is imperative that the field is ready to evaluate these targeted therapies in youth, but it is unclear if traditional JIA outcomes, which are primarily focused on measurement of peripheral disease burden, will be responsive and discriminative in this population.

In 2018, the juvenile arthritis Outcome Measures in Rheumatology (OMERACT) working group developed a core domain set prioritized by parents, patients, health care providers, researchers, and regulators.^4^ Domains were labeled as “mandatory,” “important but optional,” and “research agenda” domains. Mandatory domains included joint inflammatory signs, functional limitation, pain, patient assessment of overall well‐being, and adverse events. With the OMERACT‐recommended framework in mind, the objectives of this study were as follows: (1) to assess the responsiveness, discrimination, and minimal clinically important improvement (MCII) of multiple JIA and SpA outcome measures in a prospective observational cohort of children with axial disease; (2) to assess the potential for subgroup differences to impact these response measures; and (3) to develop a parsimonious list of measures for use in clinical trials of promising new therapies for axJSpA. To accomplish these objectives, we evaluated the measures in a prospective cohort of youth with axial disease treated with TNFi, a therapy used as standard of care and for which efficacy is anticipated for most.

## PATIENTS AND METHODS

### Participants

The protocol for this prospective study was reviewed and approved by the Institutional Review Board at Children's Hospital of Philadelphia. This was a multicenter prospective longitudinal study of youth with axJSpA evaluated in one of four pediatric hospitals between 2019 and 2024. Potentially eligible youth with JSpA were identified through the staff in the rheumatology clinics at participating centers (Children's Hospital of Philadelphia, Nationwide Children's Hospital, Children's of Alabama, and Cincinnati Children's Hospital Medical Center). Participants were aged 8 to 18 years and met the following inclusion criteria: (1) symptom onset before age 16 years, (2) fulfilled International League of Associations for Rheumatology criteria for enthesitis‐related arthritis or psoriatic arthritis^5^ or European SpA Study Group criteria,^6^ (3) biologic naïve, (4) physician diagnosis of axial arthritis (based on clinical or imaging features), and (5) clinical or imaging features prompting initiation of TNFi therapy.

### Assessments

All participants completed the baseline study visit (questionnaires and magnetic resonance imaging [MRI]). Participants without imaging evidence of sacroiliac joint (SIJ) inflammation at baseline completed questionnaires at the 12‐week visit. Participants with imaging evidence of SIJ inflammation at baseline completed questionnaires and MRI at the 12‐week visit. Participants with continued SIJ inflammation on MRI at 12 weeks completed additional questionnaires and MRI at 24 weeks. Patient‐reported outcomes were selected to include the range of domains important to patients and caregivers.^4^ Assessments included the following: Patient‐Reported Outcomes Measurement Information System (PROMIS) pediatric v2.0 short form pain interference 8a, upper extremity function 8a, mobility 8a, fatigue 10a, and pediatric scale v1.0 global health 7 (each reported as T scores with a mean of 50 and SD of 10, with higher scores representing more of the concept being measured); the Bath Ankylosing Spondylitis Functional Index (BASFI)^7^; the Bath Ankylosing Spondylitis Disease Activity Index (BASDAI)^8^; the patient global well‐being visual analog scale (VAS) (range 0–10); the patient global disease activity assessment (VAS, range 0–10); and patient pain intensity (VAS, range 0–10). At the 12‐ and 24‐week visits, participants answered the following question: “Since the start of the study, overall my SpA has: improved, stayed the same, worsened.” If the child indicated worsening or improvement, they were asked to rate the magnitude of the change as follows: not important, a little important, moderately important, or extremely important. The PROMIS short forms have been validated in children with juvenile arthritis.^9^ The interrater reliability of the BASFI was demonstrated in children and adults with juvenile‐onset SpA.^10^, ^11^

Physician‐reported metrics collected included the following: swollen joint count, tender joint count, tender enthesis count, and physician global disease activity assessment (VAS, range 0–10). Peripheral joint and tender enthesis examinations were not protocolized but included any involved joint or entheses (except the SIJ) to a maximum of 10, in accordance with how each are quantified for the clinical Juvenile Arthritis Disease Activity Score (cJADAS10) and Juvenile Spondyloarthritis Disease Activity Index (JSpADA).^3^

Overall and composite disease activity metrics collected at all study visits included the Ankylosing Spondylitis Disease Activity Score with C‐reactive protein (ASDAS‐CRP),^12^ BASDAI,^13^ JSpADA,^3^ and cJADAS.^13^ The interrater reliability of the BASDAI has been demonstrated in youth with juvenile‐onset SpA.^11^ The ASDAS‐CRP has not been validated in youth with SpA but has been validated in adults with juvenile‐onset SpA.^14^ The JSpADA, the original and modified versions, has been validated in youth with SpA.^15^, ^16^ The cJADAS has been validated in youth with juvenile arthritis.^13^ Binary response outcomes that were assessed included the pediatric the American College of Rheumatology pediatric 50%, 70%, and 90% (ACR Pedi 50, 70, 90) improvement criteria^17^; BASDAI 50% improvement^18^; Assessment of SpondyloArthritis international Society 40% improvement criteria (ASAS40)^19^; ASDAS‐CRP clinically important improvement (CII)^20^; ASDAS‐CRP major improvement (MI)^21^; and cJADAS10 minimal and inactive disease.^13^

### Imaging

MRI included a coronal oblique with STIR, a coronal oblique T1‐weighted turbo spin echo, and an axial T2‐weighted turbo spin echo with fat saturation. The central imaging team consisted of three raters with extensive experience interpreting pelvic MRI (WPM, NAC, and MLF). MRI reviews of deidentified Digital Imaging and Communications in Medicine images were completed using scoring modules on carearthritis.com and included a detailed assessment for inflammatory lesions in the SIJs using the Spondyloarthritis Research Consortium of Canada (SPARCC) SIJ inflammation score (SIS).^22^ The SIS is a validated, objective, responsive, and discriminatory measure for youth with SpA.^23^, ^24^ All raters completed recalibration exercises before assessment, and imaging was rated independently and blinded to clinical details by all three central imaging team members at the study end, agnostic to time point.

### Analysis

Demographics, clinical features, and measurements collected on the study population were summarized by mean and SD or median and interquartile range (IQR) as appropriate. Age‐ and sex‐specific z scores for weight, height, and body mass index (BMI) were calculated based on the 2000 Centers for Disease Control and Prevention (CDC) growth data for the patient's baseline visit.^25^ Overweight status was defined as a BMI ≥85th percentile as per the CDC category definitions for children and teenagers.

Responsiveness to clinical disease activity over time was assessed graphically and by mean change in scores and standardized response mean (SRM) between weeks 0 and 12. SRM was calculated by dividing the mean change between weeks 0 and 12 by the SD of the change. SRM values of 0.2 to 0.5 were considered small, values of 0.5 to 0.8 were considered medium, and values >0.8 were considered large evidence of responsiveness.

Discrimination was assessed in each measure by comparing the median change from the baseline to the 12‐week visit between participants who were “responders” and those who were “nonresponders” according to the patient assessment of global well‐being using the Wilcoxon rank sum test and standardized mean difference (SMD). SMD was calculated by dividing the mean difference between responders and nonresponders by the pooled SD of those groups. The primary definition for responders was patients with a global well‐being assessment that improved ≥2 or patients who had improvement <2 and a value of 0 at week 12; all others were considered nonresponders. Responders were secondarily defined as those who self‐reported improvement that was of at least a little importance; all others were defined as nonresponders. SMD values of 0.2 to 0.5 were considered small, values of 0.5 to 0.8 were considered medium, and values >0.8 were considered large discrimination.

#### MCII

Empirical cut points for the MCII, or the smallest change in measurement that signifies a meaningful improvement in the measure, were determined using an anchor‐based (receiver operating characteristic [ROC] curve) method using Stata package ‘cutpt.’^26^ An empirical anchor‐based approach is the primary approach recommended by the US Food and Drug Administration (FDA) for estimation of meaningful change.^27^ Patient‐reported improvement of at least “a little importance” and change in the patient‐reported global assessment of well‐being VAS of ≥2 or a follow‐up score of 0 were selected as anchors for the anchor‐based methods. Change in well‐being ≥2 was based on prior work that demonstrated this was a meaningful change.^28^, ^29^ Face validity and diagnostic test statistic performance of the different MCIIs were evaluated.

#### Measure of correlation within domains

Correlation coefficients were used to assess pairwise correlation between each of the measures to identify highly convergent measures within the same domain, adjusted for repeated measures by subject using the R package ‘rmcorr.’ Within each domain, when measures were highly correlated (r > |0.8|), those with the largest SMD were prioritized for the final list of measures for the axJSpA core domain.

#### Subgroup analysis

Differences in the core set measures’ SRMs between prespecified subgroups (sex, peripheral arthritis [yes or no], enthesitis [yes or no], and obesity status [BMI z score < or ≥85th percentile]) were determined. We also tested for differences in the proportions meeting binary response criteria (ACR 50/70/90, BASDAI 50% improvement, ASAS40, ASDAS CII and ASDAS^21^ MI, cJADAS10 minimal disease, and cJADAS10 inactive disease), stratified by the same subgroups.

### Statement of ethics and consent

The protocol for this study was reviewed and approved by a central institutional review board (IRB), the Children's Hospital of Philadelphia's Committee for the Protection of Human Subjects (IRB 19‐016713). All participating site IRBs reviewed the application and acknowledged reliance on the central IRB.

## RESULTS

The Consolidated Standards of Reporting Trials diagram is shown in Supplementary Figure S1. Seventy‐five participants enrolled, 73 (97.3%) of whom completed the baseline visit. Of the participants who completed the baseline visit, 62 (84.9%) had MRI findings consistent with axJSpA, and 57 of 62 (91.9%) completed the 12‐week visit. Participant characteristics are shown in Table 1. Of the 57 participants who completed the week 12 visit, 68.4% were male, the median age at enrollment was 15.3 years (IQR 13.5–16.9 years), 41.5% were HLA–B27 positive, 9.3% had a family history of SpA, and 49 met axJSpA classification criteria.^3^ The eight participants who failed to meet axJSpA criteria were between 5 and 28 points below the threshold. The most common reasons participants did not reach any level in a domain and received a score of zero were not meeting the unequivocal structural (n = 7) or inflammatory (n = 5) lesion definitions, no relevant family history in first‐degree relative, and HLA–B27 unknown or negative (n = 6). The axJSpA classification criteria were finalized and published after enrollment for this study was complete and were available for use as inclusion criteria. Forty‐nine (86.0%) patients were treated with adalimumab, 5 (8.8%) were treated with etanercept, 2 (3.5%) were treated with golimumab, and 1 (1.8%) was treated with infliximab. Of youth with SpA and sacroiliitis, 82.7% reported a clinical improvement of “moderate” or “large” importance to TNFi therapy by 12 weeks. Thirty‐three (58%) participants had continued MRI inflammation at 12 weeks, 76% of which reported clinical improvement of at least moderate importance (patient‐reported clinical status unavailable on three participants). Twenty‐five (75.8%) of 33 participants with continued inflammation on MRI at 12 weeks completed the 24‐week study visit. Only 12.3% of this cohort would meet current JIA trial entry criteria necessitating three or more active peripheral joints.

**Table 1:** Baseline participant characteristics*

	All participants		Participants with MRI inflammation at baseline		Participants with ≥2 MRI scans	
	n	Median (IQR) or n (%)	n	Median (IQR) or n (%)	n	Median (IQR) or n (%)
Criteria fulfilled						
ESSG	73	55 (75.3)	62	51 (82.3)	57	48 (84.2)
ILAR ERA	73	60 (82.2)	62	53 (85.5)	57	49 (86.0)
ILAR PsA	73	4 (5.5)	62	3 (4.8)	57	3 (5.3)
axJSpA	73	48 (65.8)	62	48 (77.4)	57	48 (84.2)
Age at baseline	73	15.5 (13.6–17.0)	62	15.4 (13.5–17.0)	57	15.3 (13.5–16.9)
Sex, male	73	45 (61.6)	62	41 (66.1)	57	39 (68.4)
HLA–B27 positive	66	30 (45.5)	57	25 (43.9)	53	22 (41.5)
Family history of SpA	68	9 (13.2)	58	7 (12.1)	54	5 (9.3)
BMI category, obesity	73	8 (11.0)	62	6 (9.7)	57	5 (8.8)
Polyarticular course	73	7 (9.6)	62	4 (6.5)	57	4 (7.0)
AJC ≥3	73	11 (15.1)	62	7 (11.3)	57	7 (12.3)
BMI ≥85th percentile	73	21 (28.8)	62	16 (25.8)	57	14 (24.6)
Peripheral arthritis	73	20 (27.4)	62	16 (25.8)	57	16 (28.1)
Enthesitis	73	39 (53.4)	62	33 (53.2)	57	31 (54.4)
Psoriasis	73	6 (8.2)	62	6 (9.7)	57	6 (10.5)
Inflammatory bowel disease	73	9 (12.3)	62	9 (14.5)	57	9 (15.8)
BASDAI^a^ (0–10)	72	4.7 (3.5–6.2)	61	4.5 (3.3–6.1)	56	4.4 (3.1–6.2)
JSpADA8^a^ ^,^ ^b^ (0–8)	49	3.5 (3.0–4.5)	42	3.5 (3.0–4.5)	38	3.5 (3.0–4.5)
ASDAS–CRP^a^ (0 to no upper limit)	65	2.9 (2.2–3.4)	55	2.8 (2.2–3.2)	51	2.7 (2.2–3.2)
cJADAS10^a^ (0–30)	72	9.0 (7.0–11.0)	61	9.0 (6.0–11.0)	56	9.0 (6.0–11.0)
Physician global assessment (0–10)	73	3.0 (2.0–4.0)	62	3.0 (2.0–4.0)	57	3.0 (2.0–4.0)
SPARCC sacroiliac joint inflammation score (0–72)^c^	–	–	–	–	57	11.0 (5.0–20.0)

* AJC, active peripheral joint count; ASDAS‐CRP, Ankylosing Spondylitis Disease Activity Score with C‐reactive protein; axJSpA, axial juvenile spondyloarthritis; BASDAI, Bath Ankylosing Spondylitis Disease Activity Index; BMI, body mass index; cJADAS10, clinical Juvenile Arthritis Disease Activity Score; ERA, enthesitis‐related arthritis; ESSG, European Spondyloarthropathy Study Group; ILAR, International League of Associations for Rheumatology; IQR, interquartile range; JSpADA8, 8‐item (full) Juvenile Spondyloarthritis Disease Activity Index; MRI, magnetic resonance imaging; PsA, psoriatic arthritis; SpA, spondyloarthritis; SPARCC, Spondyloarthritis Research Consortium of Canada.

^a^ Higher scores indicate more active disease.

^b^ The JSpADA index consists of 8 equally weighted measures.

^c^ Only participants with ≥2 MRI scans were reviewed by the central imaging team.

### Responsiveness

Table 2 shows the change scores and responsiveness as measured by the SRM for continuous measures from weeks 0 to 12, grouped by domains prioritized by the JIA OMERACT working group. Within the pain domain, PROMIS pain interference (0.77) and the patient pain intensity VAS (0.77) were most responsive. In the physical function domain, PROMIS mobility (0.83) and the BASFI (0.81) were most responsive. Within the joint inflammatory signs domain, the tender enthesis count (0.54) and the MRI SPARCC SIS (1.02) were most responsive. Among measures of patient perception of well‐being, the patient global well‐being VAS (0.88) and patient disease activity VAS (0.72) were most responsive. All the overall and composite disease activity response measures were responsive, but the JSpADA8 (1.28) and cJADAS10 were the largest (1.47). Box plots of change scores between responders and nonresponders are shown in Supplementary Figure S2.

**Table 2:** Change scores and SRM*

Measures	Change from index to 12 wk		
	n	Median (IQR)	SRM
Pain^a^			
PROMIS pain interference	56	−7.6 (−16.0 to 0.0)	0.77
Patient pain intensity VAS	56	−2.0 (−4.5 to 0.0)	0.77
Patient assessment of neck, back, and hip pain	56	−1.5 (−4.5 to 0.0)	0.62
Joint inflammatory signs^a^			
Active joint count	57	0.0 (0.0 to 0.0)	0.29
Tender joint count	57	0.0 (−1.0 to 0.0)	0.40
Tender enthesis count	57	0.0 (−2.0 to 0.0)	0.54
SPARCC sacroiliac joint inflammation score	57	−8.0 (−18.0 to −3.0)	1.02
Activity limitation/physical function^a^			
PROMIS upper extremity function	56	−0.0 (−0.0 to 9.3)	0.47
PROMIS mobility	56	6.1 (0.0 to 15.9)	0.83
BASFI	56	−1.1 (−2.8 to 0.0)	0.81
Patient perception of disease (well‐being)^a^			
Patient global well‐being VAS	56	−2.0 (−4.0 to −1.0)	0.88
Patient global disease activity VAS	54	−2.0 (−4.0 to 0.0)	0.72
PROMIS fatigue	56	−6.6 (−12.8 to 1.7)	0.41
PROMIS global health	56	5.1 (−0.1 to 10.3)	0.64
Overall/composite response measures			
BASDAI	56	−1.6 (−3.3 to 0.0)	0.85
JSpADA8	32	−2.0 (−3.0 to −1.0)	1.28
JSpADA7, no markers of inflammation	36	−1.5 (−2.5 to −0.5)	1.13
JSpADA7, no modified Schober's test	49	−1.5 (−2.5 to −1.0)	1.14
JSpADA6	56	−1.5 (−2.0 to −0.5)	1.05
ASDAS‐CRP	34	−1.2 (−1.9 to −0.2)	0.83
cJADAS10	56	−5.0 (−7.0 to −3.0)	1.47
Physician global assessment VAS	57	−2 (−3 to −1)	1.29

* SRM values of 0.2–0.5 were considered small, values of 0.5–0.8 were considered medium, and values >0.8 were considered large evidence of responsiveness. All raw scores generated from PROMIS instruments are translated into standardized T scores with a population mean of 50 and SD of 10. Higher scores in a domain represent more of the trait being measured; higher T scores indicate a worse outcome in the following domains: fatigue and pain interference; lower T scores indicate a worse outcome in the remaining domains. The BASDAI range is 0–10, with higher scores indicating more active disease. The BASFI range is 0–10, with higher scores indicating more impairment. All VAS ranges were 0–10, with higher scores indicating a worse state. The JSpADA index consists of 8 equally weighted measures and is scored 0–8, with higher scores indicating more active disease; the two 7‐component variants both have a score range of 0–7. ASDAS‐CRP, Ankylosing Spondylitis Disease Activity Score with C‐reactive protein; BASDAI, Bath Ankylosing Spondylitis Disease Activity Index; BASFI, Bath Ankylosing Spondylitis Functional Index; cJADAS10, clinical Juvenile Arthritis Disease Activity Score with maximum 10 active joint count; IQR, interquartile range; JSpADA6, 6‐item Juvenile Spondyloarthritis Disease Activity Index; JSpADA7, 7‐item Juvenile Spondyloarthritis Disease Activity Index; JSpADA8, 8‐item (full) Juvenile Spondyloarthritis Disease Activity Index; PROMIS, Patient‐Reported Outcomes Measurement Information System; SPARCC, Spondyloarthritis Research Consortium of Canada; SRM, standardized response mean; VAS, visual analog score.

^a^ Mandatory domain per the juvenile idiopathic arthritis Outcome Measures in Rheumatology working group.

Thirty‐seven (68.5%), 33 (61.1%), 23 (46%), and 15 (30.6%) participants met the pediatric ACR30/50/70/90 outcomes. Twenty (35.1%) and 32 (56.1%) met the cJADAS10 inactive disease and minimal disease outcomes, respectively. Twenty‐one (37.5%), 18 (50%), 4 (11.1%), and 26 (46.4%) met ASAS40, ASDAS‐CRP CII, ASDAS MI, and BASDAI 50% improvement, respectively.

### Discrimination

**Figure 1:**
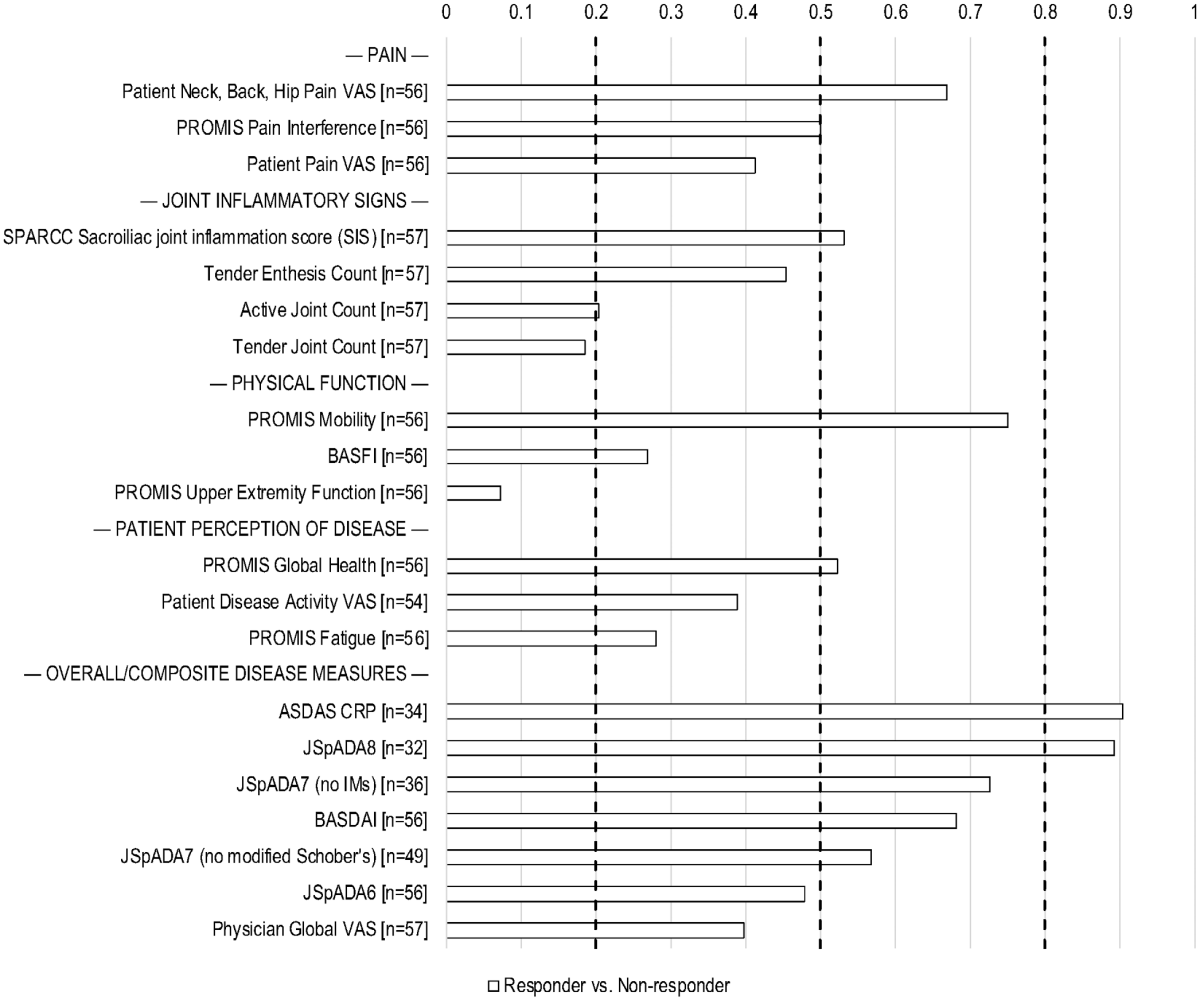
Standardized mean difference at the 12‐week visit by clinical responder status. “Clinical responder” is defined as patient global assessment VAS improvement of ≥2 or a score of 0 at week 12. Standardized mean difference values of 0.2 to 0.5 were considered small, values of 0.5 to 0.8 were considered medium, and values >0.8 were considered large evidence of discrimination. ASDAS CRP, Ankylosing Spondylitis Disease Activity Score with C‐reactive protein; BASDAI, Bath Ankylosing Spondylitis Disease Activity Index; BASFI, Bath Ankylosing Spondylitis Functional Index; IMs, markers of inflammation; JSpADA7, 7‐item Juvenile Spondyloarthritis Disease Activity Index; JSpADA8, 8‐item (full) Juvenile Spondyloarthritis Disease Activity Index; PROMIS, Patient‐Reported Outcomes Measurement Information System; SPARCC, Spondyloarthritis Research Consortium of Canada; VAS, visual analog score.

Forty participants were classified as treatment responders, and 17 participants were nonresponders. Discrimination, as measured by the SMD, for all measures is shown in Figure 1. Within the pain domain, the axial pain intensity VAS (0.67) and PROMIS pain interference (0.50) were the best discriminatory measures. In the physical function domain, PROMIS mobility (0.75) was most discriminatory, and the remainder of measures had small discrimination. Within the joint inflammatory signs domain, only the MRI SPARCC SIS (0.53) had at least medium discrimination. Among measures of patient perception of well‐being, PROMIS global health (0.52) had medium discrimination; the patient global well‐being VAS SMD was not measured against the primary responder definition because it was used to define responder status but had large SMD against the secondary responder definition (Supplementary Figure S3). Of the overall and composite disease activity measures, the JSpADA8 (0.89) and the ASDAS (0.90) had large discrimination. Both seven‐component versions of the JSpADA and the BASDAI had medium discrimination. The cJADAS10 SMD was not measured because patient global assessment, which was used to define responder status, is a component of the score. The pediatric ACR30/50/70/90, cJADAS10 inactive disease and minimal disease, and BASDAI 50% improvement all discriminated between responders and nonresponders (all *P* < 0.01).

### Correlation within domains and development of parsimonious core set

Within the pain domain, the patient pain VAS and axial pain VAS (r = 0.73) and axial pain and PROMIS pain interference (r = 0.67) had high correlation. Of these three measures, only PROMIS pain interference had a large SRM and SMD and was chosen for the core set.

In the physical function domain, the BASFI and PROMIS mobility were highly correlated (r = 0.73) and of these, only PROMIS mobility had large responsiveness and discrimination. PROMIS upper extremity had medium responsiveness but low discrimination. The PROMIS mobility measure was chosen for the core set.

Within the joint inflammatory signs domain, all the measures had low correlation, with active and tender joint counts having the highest in the domain (r = 0.45). The only measure with large responsiveness and at least medium discrimination was the SPARCC SIS. The core set for this domain includes the SPARCC SIS.

Among measures of patient perception of well‐being, patient global assessments of well‐being and disease activity were moderately correlated (r = 0.68). Both had large responsiveness, but only patient global assessment of well‐being was discriminative according to the secondary responder definition (Supplementary Figure S3). Of these measures, only the patient assessment of well‐being VAS was chosen for the core set.

All the overall and composite disease activity measures had moderate to high correlation (all except physician global assessment had r > 0.7; physician global assessment and the other measures had moderate correlation of r > 0.54). All these disease measures had high responsiveness (≥0.8) and high discrimination (≥0.8), except the physician global assessment, which had small discrimination. Collection of at least one of the overall and composite measures is recommended. The recommended core set of measures for axJSpA trials is listed in Table 3.

**Table 3:** Core set of outcome measures for axial juvenile spondyloarthritis*

Domain	Measure
Pain	PROMIS pain interference
Activity limitation/physical function	PROMIS mobility
Joint inflammatory signs	SPARCC SIS
Patient perception of disease (well‐being)	Patient assessment of well‐being VAS
Overall/composite response measures	Choose 1 of the following: JSpADA (v8 or either v7 version), cJADAS10, ASDAS‐CRP, BASDAI
Adverse events	As per investigator or funding requirement
Strongly suggested, but optional	Tender entheses count, SPARCC SIJ structural score, stiffness ≥15 min, measure of serologic inflammation

* Mandatory juvenile idiopathic arthritis domains determined based on those identified by the juvenile arthritis Outcome Measures in Rheumatology working group.^4^ ASDAS‐CRP, Ankylosing Spondylitis Disease Activity Score with C‐reactive protein; BASDAI, Bath Ankylosing Spondylitis Disease Activity Index; cJADAS10, clinical Juvenile Arthritis Disease Activity Score with maximum 10 active joint count; JSpADA, Juvenile Spondyloarthritis Disease Activity Index; PROMIS, Patient‐Reported Outcomes Measurement Information System; SIJ, sacroiliac joint; SIS, sacroiliac joint inflammation score; SPARCC, Spondyloarthritis Research Consortium of Canada; VAS, visual analog score.

### MCII

Table 4 lists the MCII for each core set measure. The anchor‐based methods using ROC analysis had the optimum combination of face validity and test statistic performance. The area under the ROC curve (AUROC) for all measures except for mobility (0.67) and SIS (0.68) was greater than 0.70. Of the overall and composite disease activity measures, the JSpADA8 had the highest AUROC, sensitivity, and specificity.

**Table 4:** Cutoffs and performance of anchor‐based MCII values in select outcome measures for patients with axial juvenile spondyloarthritis*

	MCII	AUROC	Sensitivity	Specificity
Pain				
PROMIS pain interference	−3.2	0.88	1.0	0.75
Physical function				
PROMIS mobility	3.5	0.67	0.65	0.69
Joint inflammatory signs				
SPARCC sacroiliac joint inflammation score	−5.5	0.68	0.71	0.64
Patient perception of disease				
Patient global VAS	−2.5	0.78	1.0	0.57
Overall/composite disease measures				
JSpADA8	−0.8	0.94	1.00	0.88
JSpADA7, no markers of inflammation	−0.8	0.80	0.80	0.79
JSpADA7 no modified Schober's test	−1.3	0.86	1.0	0.71
cJADAS10	−3.5	0.80	0.86	0.75
BASDAI	−1.8	0.78	1.0	0.57
ASDAS‐CRP	−0.5	0.78	0.83	0.72

* ASDAS‐CRP, Ankylosing Spondylitis Disease Activity Score with C‐reactive protein; AUROC, area under the receiver operating characteristic curve; BASDAI, Bath Ankylosing Spondylitis Disease Activity Index; cJADAS10, clinical Juvenile Arthritis Disease Activity Score with maximum 10 active joint count; JSpADA7, 7‐item Juvenile Spondyloarthritis Disease Activity Index; JSpADA8, 8‐item (full) Juvenile Spondyloarthritis Disease Activity Index; MCII, minimal clinically important improvement; PROMIS, Patient‐Reported Outcomes Measurement Information System; SPARCC, Spondyloarthritis Research Consortium of Canada; VAS, visual analog score.

### Subgroup analysis

**Figure 2:**
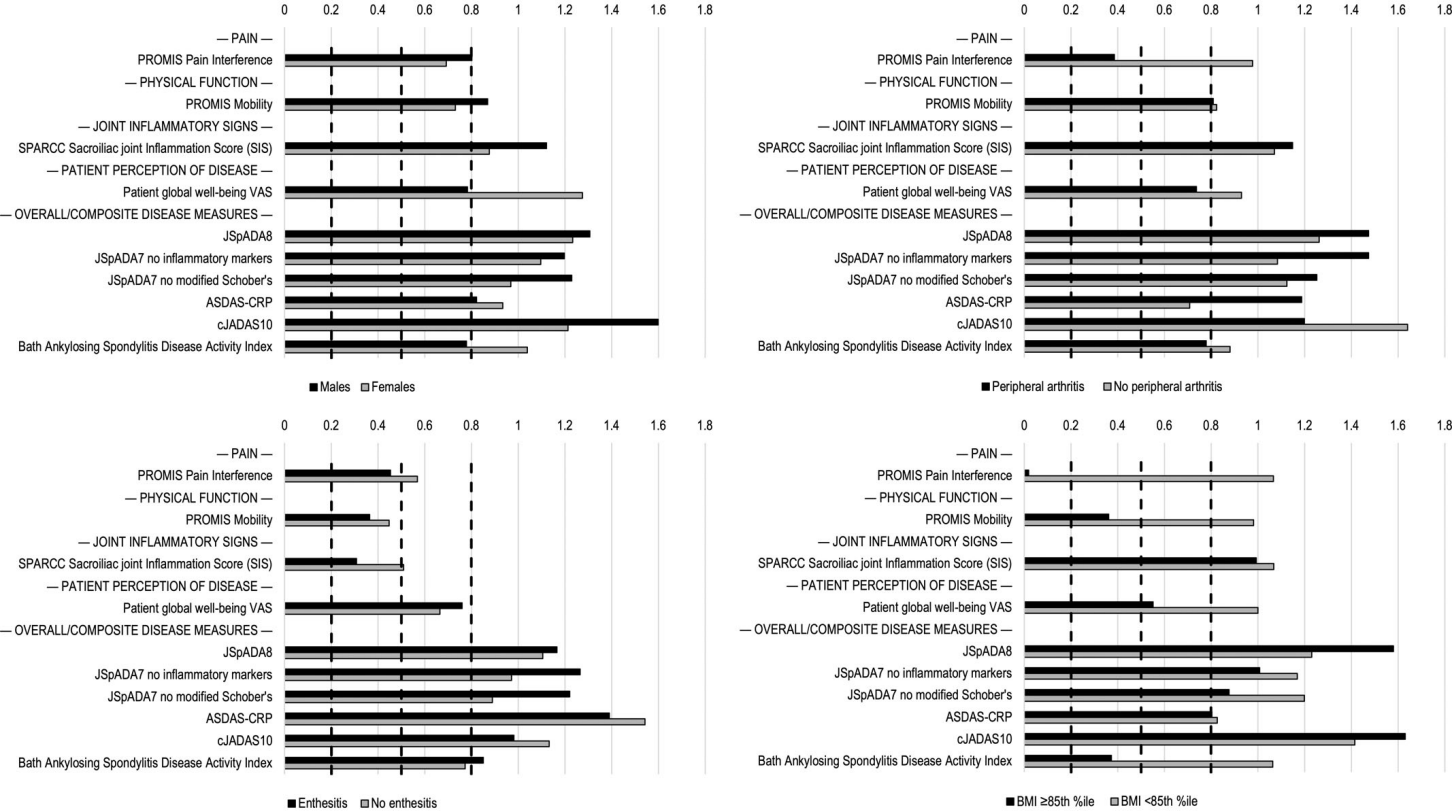
Standardized response mean by subgroup. Subgroups analyzed were the following: (top left) biologic sex (males vs females); (top right) peripheral arthritis (peripheral arthritis vs no peripheral arthritis at baseline); (bottom left) enthesitis (enthesitis vs no enthesitis at baseline), and (bottom right) BMI (≥85th percentile vs <85th percentile). The numbers of patients for each subgroup analysis are as follows: sex: male = 39, female = 18; enthesitis: yes = 31, no = 26; peripheral arthritis: yes = 16, no = 41; BMI 85th percentile: yes = 14, no = 43. ASDAS‐CRP, Ankylosing Spondylitis Disease Activity Score with C‐reactive protein; BMI, body mass index; cJADAS10, clinical Juvenile Arthritis Disease Activity Score with maximum 10 active joint count; JSpADA7, 7‐item Juvenile Spondyloarthritis Disease Activity Index; JSpADA8, 8‐item (full) Juvenile Spondyloarthritis Disease Activity Index; PROMIS, Patient‐Reported Outcomes Measurement Information System; SPARCC, Spondyloarthritis Research Consortium of Canada; VAS, visual analog score.

Responsiveness, as determined by the SRM, for prespecified subgroups for each core set of measure is shown in Figure 2. The largest differences were seen in those who were overweight (BMI ≥85th percentile) versus those with normal weight. Overweight youth had lower responsiveness than youth with normal weight for the pain, function, joint inflammatory signs and perception of disease measures and outcomes, with the most notable differences for PROMIS pain interference (0.04 vs 1.04), PROMIS mobility (0.50 vs 0.93), patient global well‐being (0.60 vs 0.97), and BASDAI (0.40 vs 1.05). Responsiveness was higher in overweight individuals for most of the overall and composite disease activity measures, most prominently for JSpADA8 (1.89 vs 1.18) and cJADAS10 (1.87 vs 1.37).

Male participants had higher responsiveness for the MRI SIS (1.12 vs 0.88) than female participants but also had higher baseline mean SIS scores (15.3 vs 10.2). Responsiveness of patient perception of disease was higher for female participants (1.28 vs 0.78). In male and female participants, responsiveness for overall disease measures and measures of pain and function were similar.

In the evaluation of those with enthesitis versus those without enthesitis, differences in responsiveness were most notable for the SPARCC SIS (0.51 vs 0.31). Responsiveness for overall disease measures weas higher in youth with enthesitis, except for the ASDAS‐CRP and cJADAS10.

In subgroup responsiveness analysis of those with and without peripheral arthritis, youth with arthritis had lower PROMIS pain interference (0.39 vs 0.98) and patient global well‐being VAS (0.74 vs 0.93), and the majority of overall disease activity measures were higher (all except cJADAS10 and BASDAI). There were no differences in responsiveness for PROMIS mobility for those with and without peripheral arthritis.

## DISCUSSION

In this prospective real‐world axJSpA population treated with TNFi, the most responsive measures over 12 weeks were the SPARCC SIS, PROMIS pain interference, the patient global well‐being VAS, and the physician global disease activity assessment. Several important key findings are worth highlighting. First, most youth with sacroiliitis, as expected, had a clinical and imaging response to TNFi therapy. Of youth with SpA and sacroiliitis, 82.7% reported a clinical improvement of “moderate” or “large” importance to TNFi therapy by 12 weeks. Of youth treated with TNFi, 70.2% met the responder definition by 12 weeks, and although 58% (33 of 57) of participants had continued MRI inflammation at 12 weeks, 77% of those patients reported clinical improvement of at least moderate importance. Second, peripheral disease (arthritis or enthesitis) occurs in only half of patients. Third, more traditional measures used to assess medication efficacy, such as active joint count and physician global disease assessment, did not perform well in this population and had either low responsiveness, low discrimination, or both. Fourth, only 12.3% of the cohort presented herein would be eligible for enrollment in a JIA trial using current inclusion criteria, which necessitate at least three active peripheral joints. Fifth, there were important subgroup differences in responsiveness, most notably in youth who were overweight versus those with normal weight. These responsiveness differences were observed for pain, mobility, and well‐being. Lastly and most importantly, there is now a parsimonious set of measures that can assess the novel efficacy of medications for this understudied condition.

The OMERACT JIA working group concluded the following domains were mandatory in JIA trials: joint inflammatory signs, functional limitation, pain, patient assessment of overall well‐being, and adverse events. In JIA trials (particularly of the polyarticular course), the joint inflammatory signs domain most commonly assesses active joint and tender joint counts. When considering JSpA, one might also consider the tender enthesis count. However, none of these three counts were responsive or discriminative in the population with axJSpA. Similar findings of low responsiveness of tender and swollen joint counts have been reported in adults with SpA, namely those with psoriatic arthritis (PsA).^30^ Youth with axJSpA, as evidenced by this cohort and others, often do not have a large (if any) burden of peripheral disease, making the lack of responsiveness not entirely surprising. The SIJs, additionally, are tricky to reliably examine because the joint does not have detectable swelling, and tenderness on examination is often nonspecific.^31^ The only measure that was both reliable and discriminative in this population for joint inflammatory signs was the MRI SPARCC inflammation score. In fact, the lack of imaging to assess TNFi efficacy at the SIJs in the earlier published trial of youth with axial disease may explain the ultimately negative result.^1^ Despite the costs of MRI, inclusion of imaging for evaluation of novel therapeutics’ effectiveness at the SIJ is imperative to carefully consider. Correlation of the MRI inflammation score and the measure used to define responder status, patient global assessment, was only moderate (0.56) because they are both measuring different but equally important concepts. In fact, the MRI inflammation score was not highly correlated with any of the other measures tested, again underscoring its importance in assessing the effectiveness of the drug at its intended target. Without imaging, dependence on measures that heavily rely on the presence of peripheral disease may result in a false‐negative conclusion. Regarding the other three mandatory OMERACT domains of functional limitation, pain, and patient assessment of overall well‐being, there were easy‐to‐administer measures that demonstrated responsiveness and discrimination for this population.

All the overall and composite disease activity measures tested, except the physician global assessment, were responsive and discriminatory. Ultimately, the choice of what, if any, composite measure to use should take into consideration the burden of peripheral disease activity at baseline in the target population, whether the drug under study is anticipated to have differential effects of certain disease manifestations (ie, peripheral arthritis, axial arthritis, dactylitis, or enthesitis), whether the trial is intended to fulfill FDA or European Medicines Agency (EMA) labeling requirements, and/or whether the measures have been validated in youth. Of the tested measures, the pediatric ACR30 is recognized as the gold standard for assessing treatment response in polyarticular course JIA and is accepted by the FDA and EMA for drug registration.^17^, ^32^ The pediatric ACR core set, cJADAS10, and JSpADA are all validated measures in youth.

Subgroup analysis demonstrated some interesting and important differences. In the subgroup analysis of youth with overweight versus those without overweight, there was a consistent and blunted response for nearly all measures among overweight individuals but most notably for PROMIS pain interference, PROMIS mobility, and global well‐being. Additionally, overweight youth started with lower or better baseline scores for all measures in comparison to normal weight youth. Differences in therapy response based on obesity status has been shown in adults with arthritis,^33^ and a negative association between BMI percentile and TNFi concentration has been shown in youth with JIA.^34^ In a meta‐analysis of select inflammatory diseases, including rheumatoid arthritis, SpA, and PsA, adults with obesity had a 60% higher odds of TNFi response failure, and the response was dose dependent, with increased odds of 6.5% with each 1‐kg/m^2^ increase in BMI.^33^ This altered response to TNFi is likely multifactorial and related to higher systemic inflammatory burden^35^, ^36^ as well as altered drug pharmacokinetics, including increased clearance, lower absorption, and alterations in the volume of distribution.^37^, ^38^ Additionally, the SRM for the MRI SIS was larger in male participants, and this may be at least partially explained by higher baseline mean SIS scores (15.3 vs 10.2). In adults with ankylosing spondylitis and nonradiographic spondylitis, sex differences have been reported frequently and include more SIJ inflammation, higher prevalence of fat lesions, and more structural damage in men compared to women.^39^, ^40^ Given these findings, consideration should be given to stratification of analysis based on BMI (≥85th percentile vs <85th percentile) if obesity is prevalent in the study population and possibly also to biologic sex if imaging is a primary outcome.

Strengths of this study include the multicenter cohort, evaluation of a comprehensive set of pediatric JIA and adult SpA outcomes, blinded central imaging scoring, and testing the responsiveness and discrimination of these measures in a real‐world setting. However, several limitations should be considered. As with any prospective study, there were some missing data, albeit minimal for all measures. Some physical examination metrics and adult SpA instruments were added to the patient questionnaires later than others, resulting in different numbers of patients for several of the adult outcomes. Evaluation for axial disease in this cohort was done per the treating physician. Because thresholds to initiate this evaluation may differ across centers and by provider, asymptomatic and mild cases may have been missed. However, those with milder cases are unlikely to be the patients who would be considered for initiation of biologic therapy and/or candidates for a pragmatic therapy trial for axial disease.

In conclusion, most youth with axJSpA had a clinical and imaging response to TNFi therapy. Some of the most responsive and discriminative measures in this population were not the standard outcomes for current polyarticular course JIA trials. Moreover, this study's results suggest that standard calculated response metrics used in pediatric clinical trials may underestimate clinical response in children with axial disease, and subgroup analysis demonstrated some interesting differences regarding overweight status and biologic sex that warrant consideration in the design of future trials. Lastly, and most importantly, there is now a parsimonious set of measures that can assess the efficacy of novel medications for this understudied condition.

## AUTHOR CONTRIBUTIONS

All authors contributed to at least one of the following manuscript preparation roles: conceptualization AND/OR methodology, software, investigation, formal analysis, data curation, visualization, and validation AND drafting or reviewing/editing the final draft. As corresponding author, Dr Weiss confirms that all authors have provided the final approval of the version to be published and takes responsibility for the affirmations regarding article submission (eg, not under consideration by another journal), the integrity of the data presented and the statements regarding compliance with institutional review board/Declaration of Helsinki requirements.

## Supporting information


**Disclosure form**.

**Appendix S1:** Supplementary Information
