# Supplementary material for: Core Set of Responsive and Discriminatory Measures for Use in Pragmatic Trials of Youth With Axial Juvenile Spondyloarthritis
Source: Arthritis Care Res (Hoboken). 2025 Jun 23;77(10):1229–39. doi: 10.1002/acr.25565 (PMC12353632; doi:10.1002/acr.25565)
Supplement: Supplementary file 2 — Appendix S1: Supplementary Information [file ACR-77-1229-s002.docx]

**Supplement**

**Figure S1. CONSORT diagram**


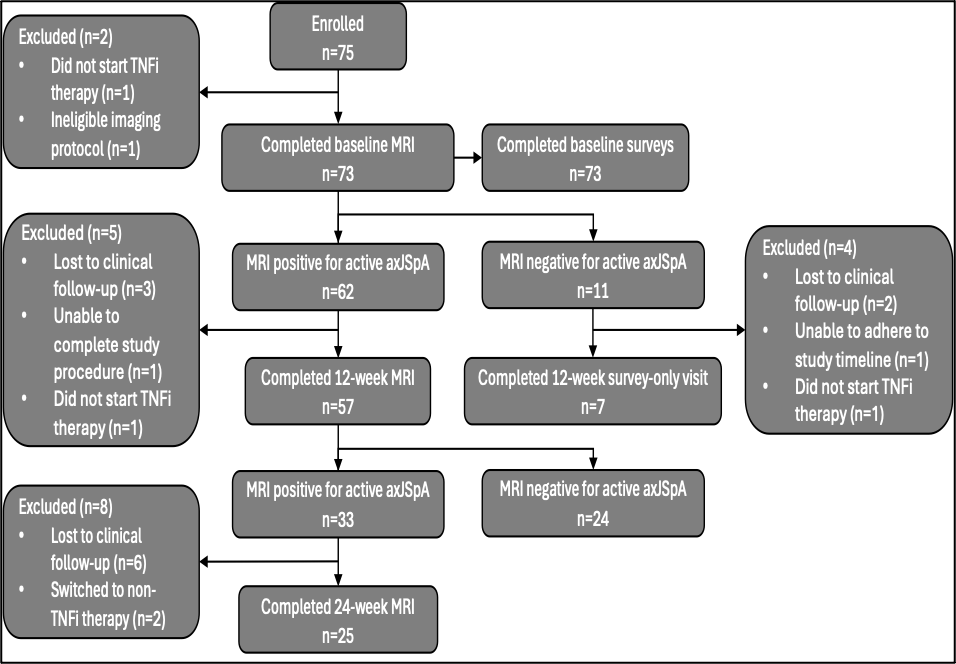


| A | 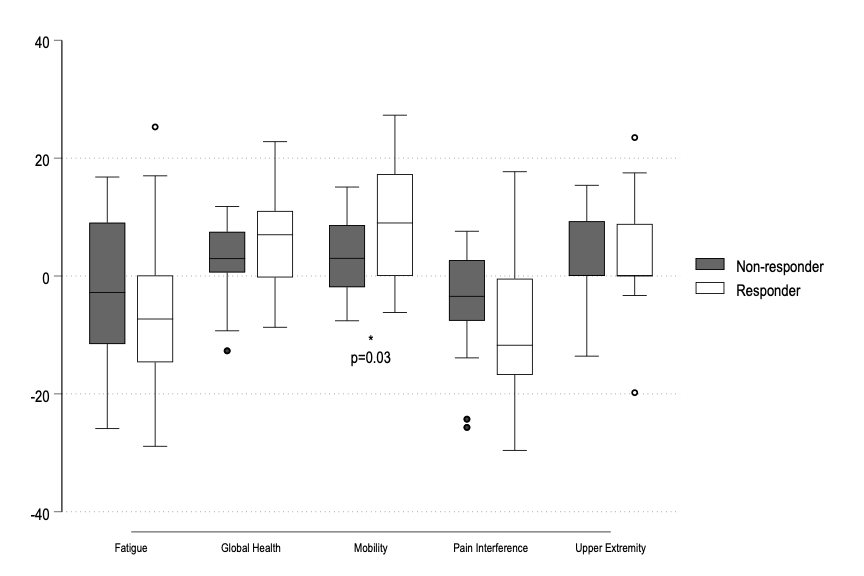 |
| --- | --- |
| B | 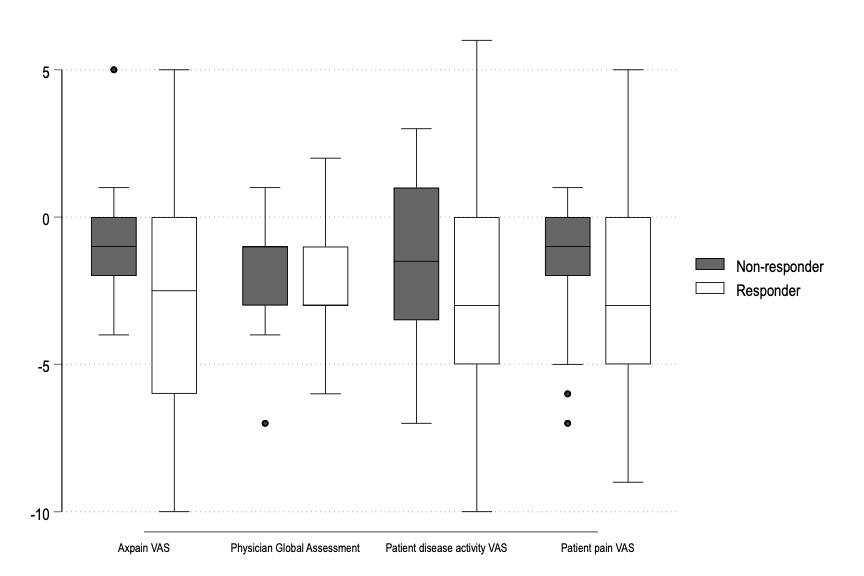 |
| C | 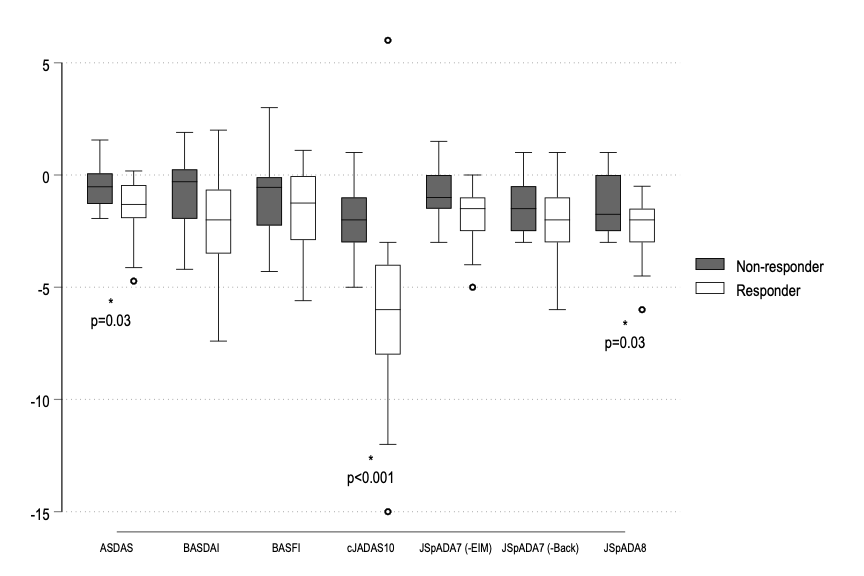 |

**Figure S2. Change scores (unadjusted) between patient responders and non-responders**.

Legend. Change scores by patient responder status for: A) PROMIS Fatigue, Global Health, Mobility, Pain interference and upper extremity function; B) visual analogue scales for neck/back/hip pain, patient-reported disease activity, patient-reported pain, C) composite disease activity measures including ASDAS CRP, BASDAI, BASFI, cJADAS10, JSpADA-8, JSpADA-7 (EIM; no labs), JSpADA-7 (-back; no modified Schober’s)

**Figure S3. Discrimination by responder status defined as self-reported improvement that was of at least a little importance.**
